# Temperature-, pH- and CO_2_-Sensitive Poly(*N*-isopropylacryl amide-*co*-acrylic acid) Copolymers with High Glass Transition Temperatures

**DOI:** 10.3390/polym8120434

**Published:** 2016-12-14

**Authors:** Yeong-Tarng Shieh, Pei-Yi Lin, Tao Chen, Shiao-Wei Kuo

**Affiliations:** 1 Department of Chemical and Materials Engineering, National University of Kaohsiung, Kaohsiung 81148, Taiwan; m033100007@student.nsysu.edu.tw; 2 Department of Materials and Optoelectronic Science, National Sun Yat-Sen University, Kaohsiung 80424, Taiwan; 3 Ningbo Institute of Material Technology and Engineering, Key Laboratory of Graphene Technologies and Applications of Zhejiang Province, Chinese Academy of Science, Zhongguan West Road 1219, Ningbo 315201, China; tao.chen@nimte.ac.cn

**Keywords:** temperature-sensitive, pH-sensitive, CO_2_-sensitive, hydrogen bonding, copolymers

## Abstract

A series of poly(*N*-isopropylacrylamide-*co*-acrylic acid) (PNIPAAm-*co*-PAA) random copolymers were synthesized through free radical copolymerization in MeOH. The incorporation of the acrylic acid units into PNIPAAm tended to enhance the glass transition temperature (*T*_g_), due to strong intermolecular hydrogen bonding between the amide groups of PNIPAAm and the carboxyl groups of PAA, as observed using ^1^H nuclear magnetic resonance (NMR) and Fourier transform infrared (FTIR) spectroscopic analyses. The lower critical solution temperature (LCST) increased upon increasing the pH of the aqueous solution containing PNIPAAm-*co*-PAA because the COOH groups of the PAA segment dissociated into COO^−^ groups, enhancing the solubility of the copolymer. In addition, high-pressure differential scanning calorimetry revealed that the LCSTs of all the aqueous solutions of the copolymers decreased upon increasing the pressure of CO_2_, suggesting that CO_2_ molecules had displaced H_2_O molecules around the polar CONH and COOH groups in PNIPAAm-*co*-PAA, thereby promoting the hydrophobicity of the copolymers in the aqueous solution. In addition, the values of *T*_g_ of a film sample increased upon treatment with supercritical CO_2_, implying that intermolecular interactions in the copolymer had been enhanced after such treatment.

## 1. Introduction

Increasing the glass transition temperatures (*T*_g_) of polymeric materials is interesting in polymer science due to the strong economic incentives arising from their potential industrial applications [1,2,3,4]. In general, values of *T*_g_ are strongly dependent on the chemical and physical nature of polymeric materials, including their molecular weights, numbers of bulky groups, degrees of branching, degrees of crosslinking, and strength of intermolecular interactions [5,6,7,8,9]. In previous studies [10,11,12,13], we found that copolymerization of two monomers possessing strong hydrogen bonding donor or acceptor units can lead to significant enhancements in glass transition temperatures, due to so-called “compositional heterogeneity” [14].

Smart materials exhibit changes in their properties that are dependent on their environment. For example, they could shrink or swell in response small variations in pH, temperature, or ionic strength [15,16]. Poly(*N*-isopropylacrylamide) (PNIPAAm) is a well-known smart material that undergoes a sharp coil-to-globule transition in aqueous solution at approximately 32 °C (its lower critical solution temperature (LCST)), from a hydrophilic (hydrated) state below this temperature to a hydrophobic (dehydrated) state above it [17,18]. Poly(acrylic acid) (PAA) also undergoes phase transitions in aqueous solution that are dependent on pH, allowing it to be used in site-specific drug delivery [19]. Combinations of PNIPAAm and PAA in random copolymers not only result in dual stimuli-responsive behavior (temperature and pH) in aqueous solution [20,21,22] but also significant increases in values of *T*_g_ in response to strong intermolecular hydrogen bonding in the bulk state.

In addition, the carbonyl (C = O) groups in PNIPAAm and PAA polymers are capable of interacting with CO_2_ molecules through the dipole–dipole interactions [23,24]. The CO_2_ molecules can around the C = O groups in PNIAAm and PAA in aqueous solution, thereby displacing H_2_O molecules from around the polar CONH and COOH groups of PNIPAAm and PAA, respectively. This phenomenon may lead to a decrease in the LCST of PNIAAm-*co*-PAA copolymers in aqueous solution, due to increased hydrophobicity. In this study, we prepared a series of PNIAAm-*co*-PAA random copolymers through free radical copolymerization in MeOH solution. Although PNIAAm-*co*-PAA random copolymers have been studied previously as superhydrophobic surfaces, hydrogels, nanoparticles, and microgels for drug delivery [25,26,27,28], to the best of our knowledge their thermal properties, hydrogen bonding, and LCST behavior under CO_2_ atmosphere or after CO_2_ treatment have not been investigated. Therefore, the copolymer compositions, hydrogen bonding interactions, thermal properties, and LCST behavior of PNIAAm-*co*-PAA random copolymers were characterized in this study using ^1^H nuclear magnetic resonance (NMR) spectroscopy, Fourier transform infrared (FTIR) spectroscopy, differential scanning calorimetry (DSC), and high-pressure differential scanning calorimetry (HP-DSC) analyses.

## 2. Experimental

### 2.1. Materials

**Scheme 1:**
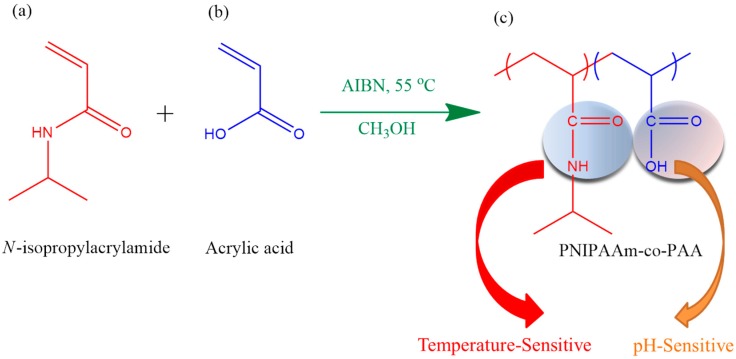
Synthesis of temperature-, pH- and CO_2_-responsive PNIPAAm-*co*-PAA copolymers. Chemical structures of the: (**a**) *N*-isoproylacrylamide monomer; (**b**) acrylic acid monomer; and (**c**) PNIPAAm-*co*-PAA copolymers.

*N*-Isopropylacrylamide (NIPAAm) was purchased from Tokyo Kasei (Tokyo, Japan) and recrystallized from *n*-hexanes. Acrylic acid (AA) was obtained from the Alfa Aesa (Heysham, Lancashire, UK). All solvents were obtained from Sigma-Aldrich (St. Louis, MO, USA) and used as received. PNIPAAm-*co*-PAA copolymers were synthesized from different monomer molar ratios through free radical copolymerization using azobis(isobutyronitrile) as the initiator in MeOH under a N_2_ atmosphere (Scheme 1). The 2 M monomer mixture containing 0.05 M AIBN was stirred at 55 °C for 4 h in a circulated thermostat water bath. For purification of the pure PNIPAAm homopolymer, the product was dissolved in MeOH at room temperature and then added into deionized water at 45 °C, causing precipitation of the PNIPAAm homopolymer [29]. This dissolution/precipitation procedure was repeated three times and then the product was dried in a vacuum oven at 60 °C for 1 day. For purification of the pure PAA homopolymer, the reaction mixture was concentrated and then was ether added to precipitate the PAA. The purified PAA homopolymer was obtained following vacuum drying for 1 day. To purify the PNIPAAm-*co*-PAA copolymers, the products were dissolved in MeOH and then ether was added to precipitate the copolymers. The settled products were dried in an oven; the dried precipitates were dissolved in 0.15 M aqueous NaOH and heated at 60 °C to precipitate PNIPAAm, which was filtered off. The filtrates were treated with a few drops of concentrated HCl and then heated at 60 °C to precipitate the PNIPAAm-*co*-PAA copolymers. The purified PNIPAAm-*co*-PAA copolymers were washed with ether until neutral and then dried in a vacuum oven for 1 day.

### 2.2. Characterization of PNIPAAm-co-PAA Copolymers in Aqueous Solutions and Films

The molecular weights and polydispersity indices (PDIs) of the synthesized PNIPAAm-*co*-PAA copolymers were determined using a Waters 510 gel permeation chromatography (GPC) system equipped with a refractive index detector and three Ultrastyragel columns (100, 500 and 1000 Å) connected in series. *N*,*N*-Dimethylformamide (DMF) was the eluent, at a flow rate of 0.8 mL/min, at 30 °C. A Bruker Tensor-27 (Billerica, MA, USA) FTIR spectrometer was used to quantitatively characterize the hydrogen bonding interactions in the copolymers, which were cast from THF solutions onto KBr crystal plates. The spectra were collected at a resolution of 4 cm^−1^ and a sensitivity of 32 scans at room temperature. A Varian Unity Inova-500MHz ^1^H NMR spectrometer (McKinley Scientific, Sparta, NJ, USA) was used to quantitatively characterize the copolymers’ compositions and hydrogen bonding interactions when dissolved in DMSO-*d*_6_. The LCST behavior of the PNIPAAm-*co*-PAA copolymers was measured from plots of their visible light transmittance at 550 nm with respect to temperature, using 5 wt % copolymer aqueous solutions at various values of pH. The inflection point of the transmittance–temperature curve was assigned as the LCST of the copolymer aqueous solution. A Q100 DSC apparatus (TA Instrument, New Castle, DE, USA) was also used to determine the LCSTs of the 5 wt % copolymer aqueous solutions at various values of pH. The DSC system was operated with heating at 2 °C/min from 10 to 55 °C and then cooling at 2 °C/min to 10 °C, followed by heating again at 2 °C/min to 55 °C to record the endothermic peak (i.e., the LCST). A Q20 HP-DSC apparatus (TA Instruments) was used to investigate the effect of a CO_2_ atmosphere on the LCST of 5 wt % aqueous solutions of the PNIPAAm-*co*-PAA copolymers at various values of pH. The chamber was first purged with CO_2_ for 5 min before heating under CO_2_ from 16 to 45 °C at a rate of 2 °C/min. The peak temperature and area of the endothermic peak in the first heating curve of each sample were recorded and taken as the LCST and enthalpy, respectively. The glass transition temperatures (*T*_g_) of the PNIPAAm-*co*-PAA copolymers of various compositions were determined after casting them from aqueous solutions at various values of pH. They were measured through DSC, with an initial heating and cooling cycle at 20 °C/min between 40 and 200 °C and then with heating again at 10 °C/min to 200 °C. The CO_2_-dependence of the values of *T*_g_ of the cast films of the various copolymer compositions was also investigated after treatment in supercritical carbon dioxide fluid (scCO_2_) at 2000 psi and 32 °C for 1 h, with a depressurization time of 1 h.

## 3. Results and Discussion

### 3.1. Synthesis of PNIPAAm-co-PAA Random Copolymers

**Figure 1:**
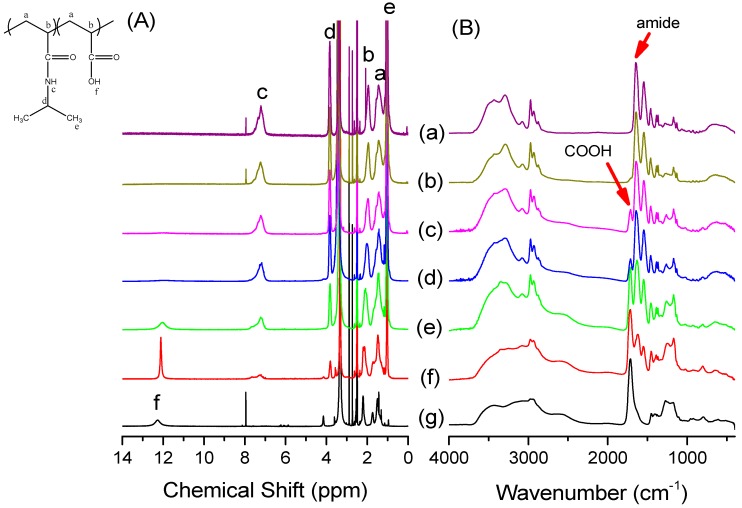
(**A**) ^1^H NMR; and (**B**) FTIR spectra of: (a) pure PNIPAAm; (b) PNIPAAm97-*co*-PAA3; (c) PNIPAAm87-*co*-PAA13; (d) PNIPAAm80-*co*-PAA20; (e) PNIPAAm57-*co*-PAA43; (f) PNIPAAm30-*co*-PAA70; and (g) pure PAA.

**Figure 2:**
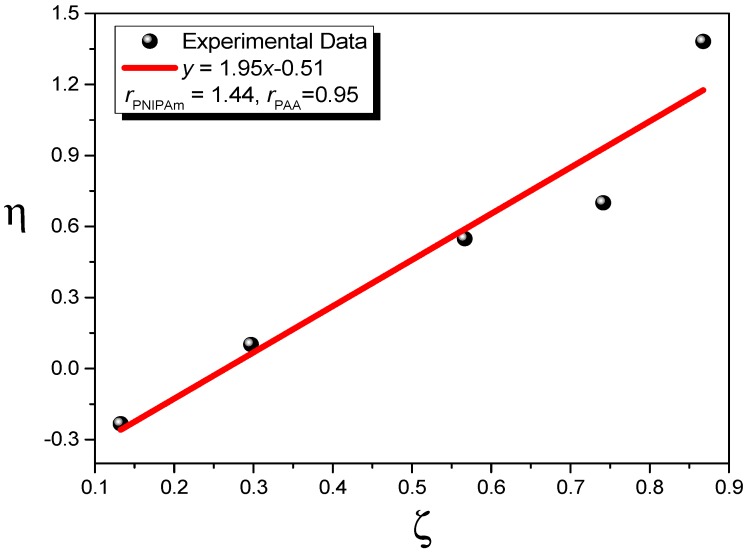
Kelen–Tudos plots of PNIPAAm-*co*-PAA random copolymers.

We synthesized a series of PNIPAAm-*co*-PAA random copolymers through free radical copolymerization in MeOH solution (Scheme 1). The compositions of these PNIPAAm-*co*-PAA random copolymers were determined using ^1^H NMR and FTIR spectroscopy. Figure 1A displays the ^1^H NMR spectra of PNIPAAm-*co*-PAA random copolymers recorded as DMSO-*d*_6_ solutions. For the pure PNIPAAm homopolymer, the signals for the CH and CH_2_ groups on the main chain were located between 1.46 and 1.96 ppm. The other alkyl CH and CH_3_ protons for pure PNIPAAm appeared as multiplets at 3.86 and 1.02 ppm, respectively; a singlet at 7.21 ppm represented the proton of the amide group (CONH). Similarly, for the pure PAA homopolymer, the CH and CH_2_ groups on the main chain were located between 1.46 and 2.19 ppm, with a singlet at 12.26 ppm corresponding to the proton of the carboxyl group (COOH). The compositions of PAA in copolymers were determined from the peak ratio of *A*_COOH_/(*A*_COOH_ + *A*_CONH_), based on the signals 12.26 and 7.21 ppm. The compositions of these PNIPAAm-*co*-PAA random copolymers were confirmed using FTIR spectroscopy (Figure 1B). For pure PNIPAAm, the two peaks at 1644 and 1544 cm^−1^ represented the amide I and amide II stretching vibration modes; for pure PAA, the characteristic band at 1710 cm^−1^ represented the self-association hydrogen-bonded COOH groups. Clearly, the ratio of the signal area at 1710 cm^−1^ (COOH) with respect to the amide I (CONH) peak increased upon increasing the PAA content in the PNIPAAm-*co*-PAA random copolymers. Table 1 summarizes the feed ratios of the NIPAAm/AA monomers and the resultant copolymer compositions determined based on ^1^H NMR spectroscopy. The reactivity ratios were determined using the Kelen and Tudos methodology, as we have described previously [30,31]. Figure 2 displays the results graphically; for the PNIPAAm-*co*-PAA copolymers, we determined values of *r*_PNIPAAm_ and *r*_PAA_ of 1.44 and 0.95, respectively, suggesting a tendency for ideal random copolymerization.

### 3.2. Thermal Properties of PNIPAAm-co-PAA Random Copolymers

**Figure 3:**
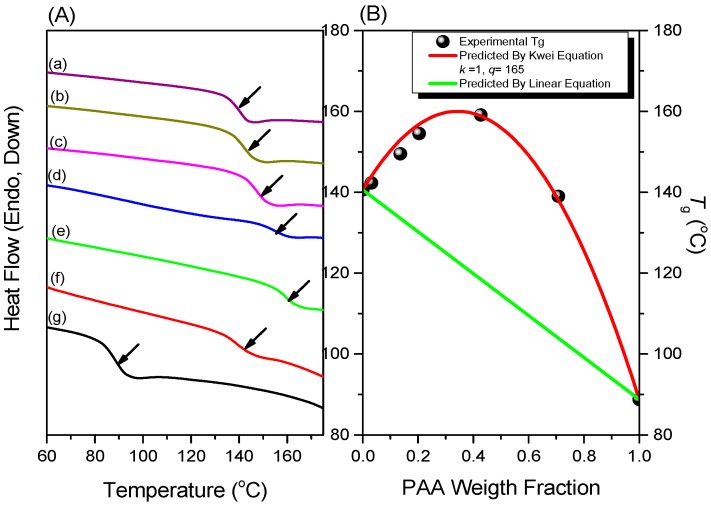
(**A**) DSC thermograms of PNIPAAm-*co*-PAA random copolymers: (a) pure PNIPAAm; (b) PNIPAAm97-*co*-PAA3; (c) PNIPAAm87-*co*-PAA13; (d) PNIPAAm80-*co*-PAA20; (e) PNIPAAm57-*co*-PAA43; (f) PNIPAAm30-*co*-PAA70; and (g) pure PAA. (**B**) Experimental values of *T*_g_, with lines predicted by the Kwei and linear equations.

Figure 3A displays DSC thermograms of pure PNIPAAm, pure PAA, and various PNIPAAm-*co*-PAA random copolymers, recorded at temperatures ranging from 60 to 180 °C. The pure PNIPAAm and pure PAA provided values of *T*_g_ of approximately 140 and 88 °C, respectively; each of the PNIPAAm-*co*-PAA random copolymers provided a single value of *T*_g_ in the range 140–159 °C, significantly higher than those of the homopolymers. This large positive deviation in the behavior of the glass transition temperatures suggests the presence of strong intermolecular interactions between the two polymer segments.

**Scheme 2:**
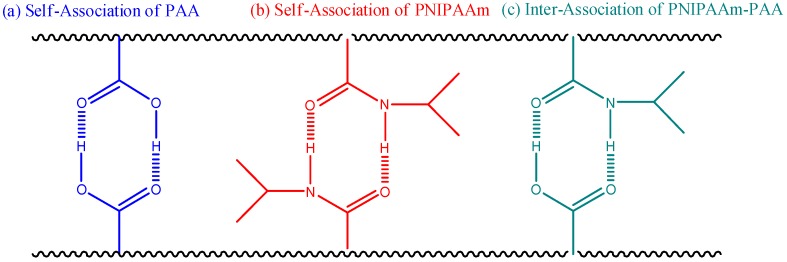
Noncovalent interactions of PNIPAm-*co*-PAA copolymers: (**a**) self-association of PAA units; (**b**) self-association of PNIPAAm units; and (**c**) inter-association of PNIPAAm-*co*-PAA.

The Kwei equation is commonly adopted to explain the behavior of the values of *T*_g_ of system displaying strong intermolecular interactions [7]:

(1) $$T_{g}=\frac{W_{1}T_{g1}+kW_{2}T_{g2}}{W_{1}+kW_{2}}+qW_{1}W_{2}$$

where *T*_gi_ and *W*_i_ represent the glass transition temperature and weight fraction of each component *i*; and *k* and *q* are both fitting constants describing the strength of the intermolecular interactions. Using this equation (red line in Figure 3B), we calculated values of *k* and *q* of 1 and 160, respectively. This positive value of *q* (higher than the linear rule, green line) suggests that intermolecular hydrogen bonding between the PNIPAAm and PAA segments (Scheme 2c) in the PNIPAAm-*co*-PAA random copolymers was stronger than the self-association hydrogen bonding of either the PNIPAAm segments (Scheme 2b) or the PAA segments (Scheme 2a).

**Figure 4:**
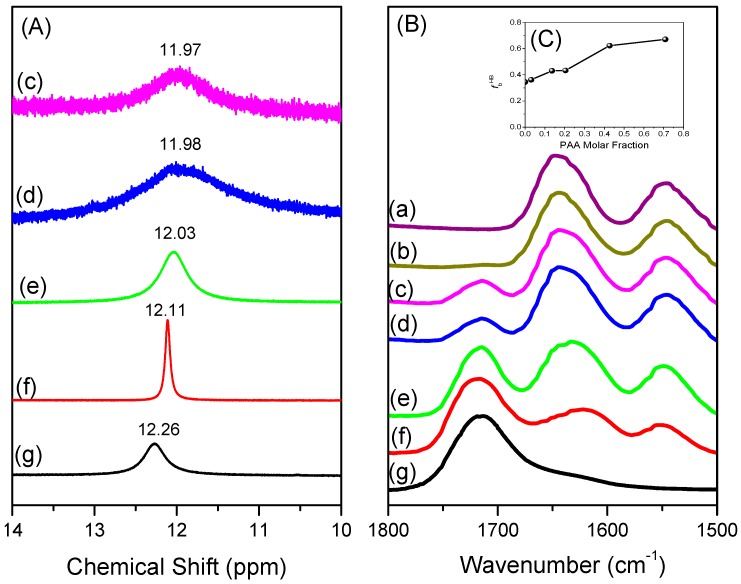
(**A**) ^1^H NMR spectra, presented in the range 14–10 ppm, displaying the signal of the proton of the acrylic acid units. (**B**) FTIR spectra in the range 1800–1500 cm^−1^ displaying the COOH and CONH absorptions of: (a) pure PNIPAAm; (b) PNIPAAm97-*co*-PAA3; (c) PNIPAAm87-*co*-PAA13, (d) PNIPAAm80-*co*-PAA20; (e) PNIPAAm57-*co*-PAA43; (f) PNIPAAm30-*co*-PAA70; and (g) pure PAA. (**C**) Fractions of inter-associated hydrogen bonds between the PNIPAAm and PAA segments for various PAA molar fractions.

**Figure 5:**
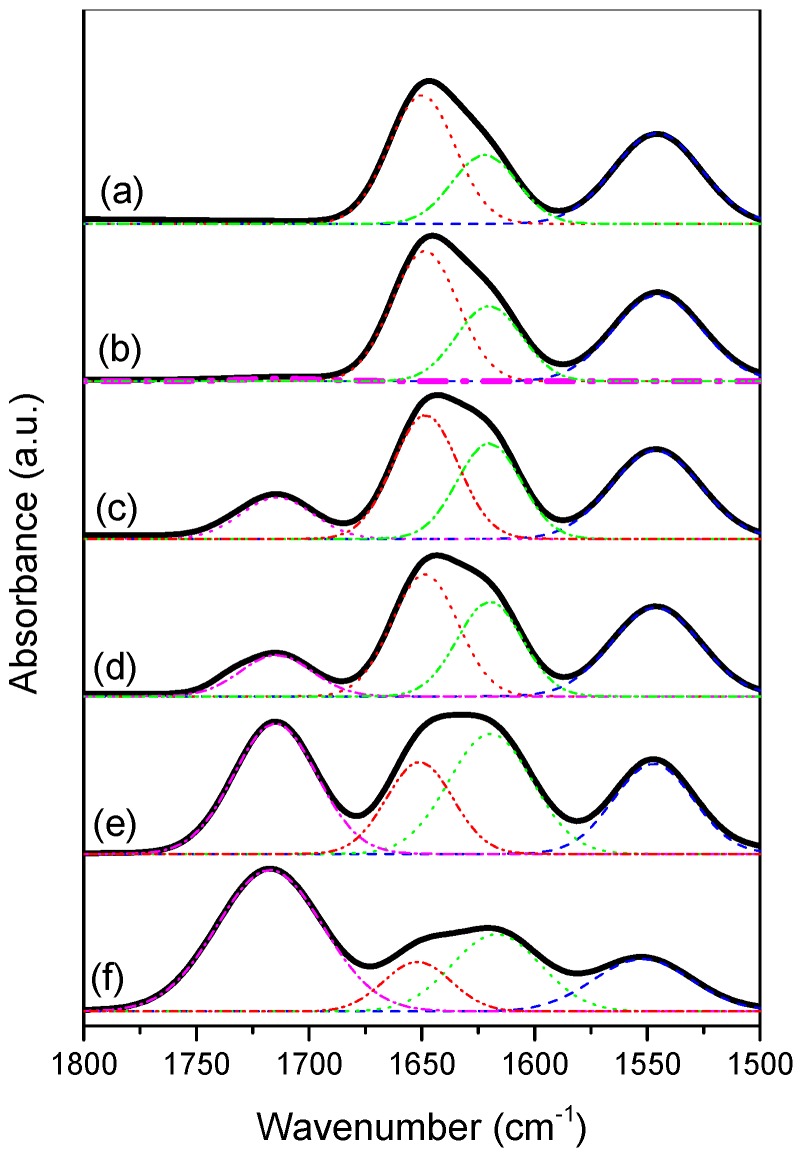
Curving fitting of data from FTIR spectra (dash lines are curve fitting results) of: (a) pure PNIPAAm; (b) PNIPAAm97-*co*-PAA3; (c) PNIPAAm87-*co*-PAA13; (d) PNIPAAm80-*co*-PAA20; (e) PNIPAAm57-*co*-PAA43; and (f) PNIPAAm30-*co*-PAA70.

NMR and FTIR spectroscopy are two highly effective methods for characterizing intermolecular interactions. Figure 4A presents ^1^H NMR spectra of various PNIPAAm-*co*-PAA random copolymers in DMSO-*d*_6_ solution. The proton of the COOH groups from the PAA segments underwent a high-field shift from an initial value of 12.26 ppm for pure PAA to 11.97 ppm for PNIPAAm87-*co*-PAA13, consistent with intermolecular hydrogen bonding between the CONH and COOH groups in PNIPAAm-*co*-PAA random copolymers (Scheme 2c) [32]. Figure 4B illustrates the FTIR spectral region of the C=O groups of the PNIPAAm-*co*-PAA random copolymers. The spectrum of pure PNIPAAm displays two peaks at 1644 and 1544 cm^−1^, corresponding to the amide I and amide II stretching vibration modes; for pure PAA, a characteristic band appeared at 1710 cm^−1^, corresponding to the self-association hydrogen-bonded COOH groups. Upon increasing the PAA composition in the PNIPAAm-*co*-PAA random copolymers, the signal for the amide I groups of PNIPAAm split into two bands, which we assigned to the free amide I groups at 1644 cm^−1^ and the intermolecular hydrogen bonded amide groups at 1615 cm^−1^ (Scheme 2c). These two absorption bands were resolvable using the Gaussian function (Figure 5); the fractions of intermolecular hydrogen-bonded amide group are summarized in Figure 4C (insets to Figure 4B). The fraction of hydrogen-bonded amide I groups increased upon increasing the PAA composition in the random copolymers. These strong intermolecular hydrogen bonding interactions presumably enhanced the glass transition temperatures of the PNIPAAm-*co*-PAA random copolymers, as determined from the DSC analyses.

### 3.3. LCST Behaviors of PNIPAAm-co-PAA Random Copolymers

**Figure 6:**
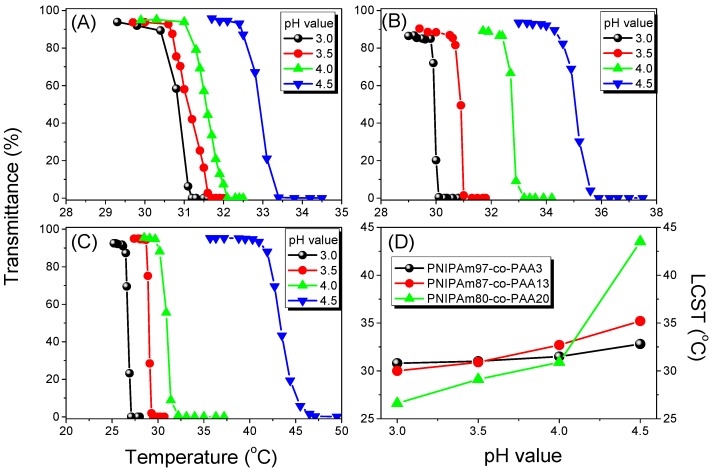
(**A**–**C**) Transmittance, based on UV–Vis spectra, at 550 nm of aqueous solutions at various temperatures and values of pH for: (**A**) PNIPAAm97-*co*-PAA3; (**B**) PNIPAAm87-*co*-PAA13; and (**C**) PNIPAAm80-*co*-PAA20 random copolymers. (**D**) Corresponding LCSTs of aqueous PNIPAAm-*co*-PAA solutions at various values of pH.

**Figure 7:**
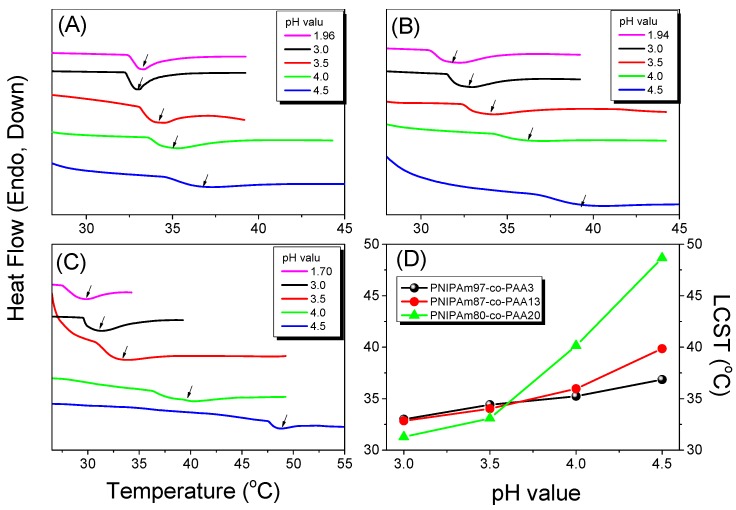
(**A**–**C**) DSC thermograms of aqueous solutions at various values of pH for: (**A**) PNIPAAm97-*co*-PAA3; (**B**) PNIPAAm87-*co*-PAA13; and (**C**) PNIPAAm80-*co*-PAA20 random copolymers. (**D**) Corresponding LCSTs of aqueous PNIPAAm-*co*-PAA solutions at various values of pH.

In general, the random coils of PNIPAAm chains in aqueous solution collapse to form dense globule chains at approximately 32 °C. Our PNIPAAm-*co*-PAA random copolymers, however, displayed dual temperature- and pH-responsive behavior in aqueous solution, with the transparent aqueous solutions becoming opaque at temperatures above a specific temperature and at values of pH above a specific pH, as revealed in Figure 6 from UV–Vis spectroscopic analyses. The opaque solution reverted into a transparent solution again when the temperature decreased, characterizing a reversible phase transition. In addition, the LCSTs increased upon increasing the pH for all of our PNIPAAm-*co*-PAA random copolymers (Figure 6A–C), presumably because of their better solubility in aqueous solutions at higher values of pH; Figure 6D summarizes the results. We also used the DSC first heating scan to determine the LCST behavior for all of our PNIPAAm-*co*-PAA random copolymers in aqueous solutions at various values of pH (Figure 7). The obvious endothermic peaks in all of the heating curves corresponded to coil-to-globule phase transitions, from dissolved to turbid states, of the polymer chains in the aqueous solutions [33,34]. We observed a trend in the LCSTs similar to that determined spectroscopically: the values increased upon increasing the pH for all of our PNIPAAm-*co*-PAA random copolymers (Figure 7A–C); Figure 7D summarizes the results. We found, however, that the LCSTs determined using DSC were all higher than those obtained using UV–Vis spectroscopy, by approximately 2–5 °C, at the same PAA composition in the random copolymer and at the same pH. This apparent discrepancy in the LCSTs from the DSC and UV spectroscopic analyses can be explained by considering the different experimental conditions: we would expect the dynamic measurements from heating scans in DSC analyses to provide higher LCSTs than would the static measurements from heating scans of UV–Vis spectroscopic analyses.

**Scheme 3:**
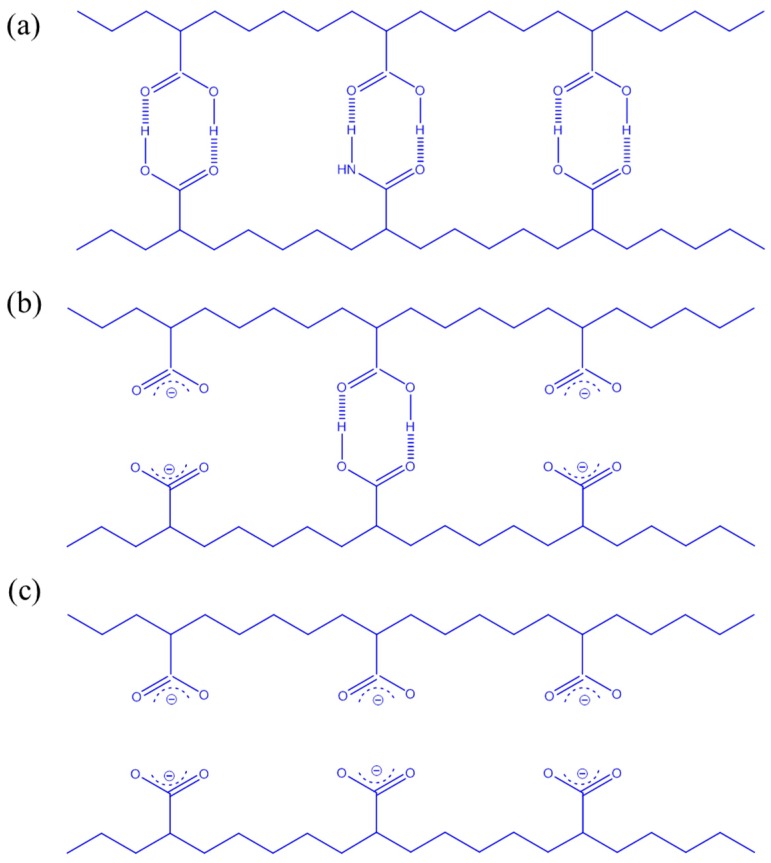
Possible PAA chain behavior in PNIPAAm-*co*-PAA copolymers at values of pH of: (**a**) 3.0; (**b**) 3.5–4.0; and (**c**) 4.5.

**Figure 8:**
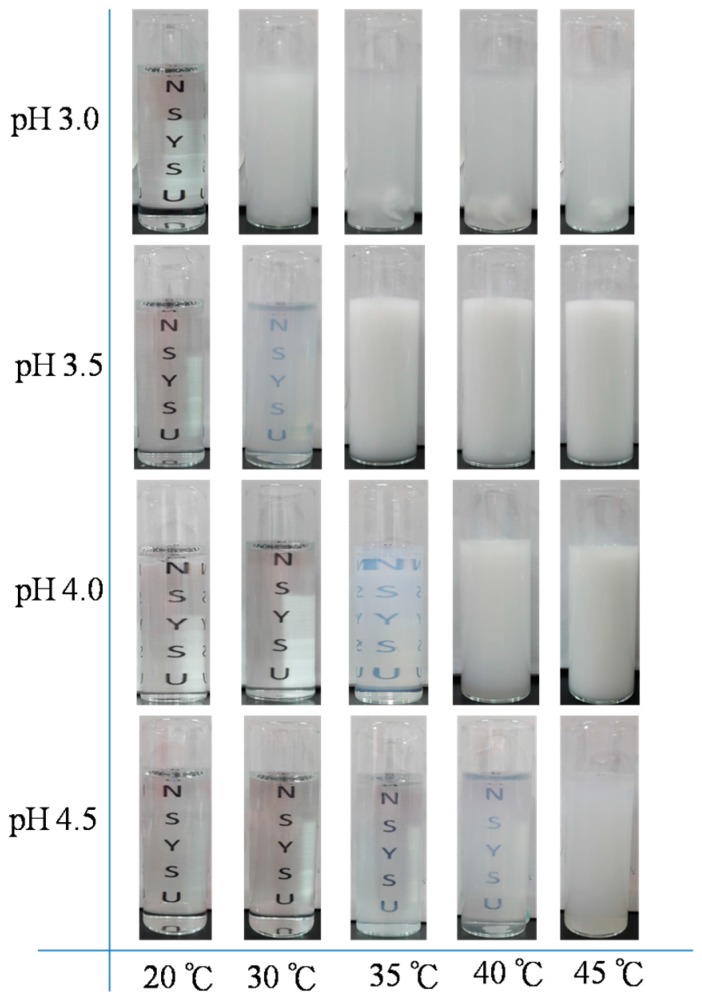
Photographs of the random copolymer PNIPAAm80-*co*-PAA20 in aqueous solutions at various temperatures and values of pH.

**Scheme 4:**
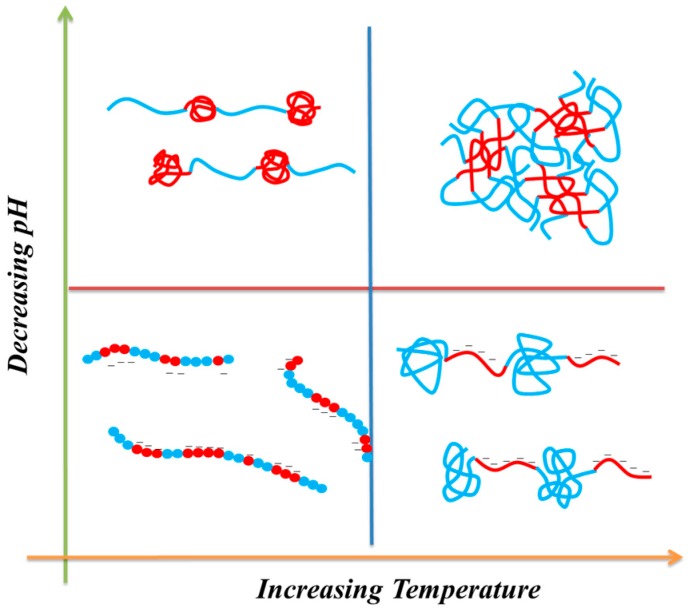
Possible chain behavior of PNIPAAm-*co*-PAA copolymers in aqueous solutions at various temperatures and values of pH (red color is PNIPAAm segment and blue color is PAA segment).

On the basis of the UV–Vis spectroscopic and DSC analyses, we found that the LCSTs increased upon increasing the pH for all PNIPAAm-*co*-PAA random copolymers. This behavior can be attributed to the enhanced solubility in aqueous solution arising from dissociation of the COOH groups of the PAA segments (Scheme 3). At lower PAA compositions (i.e., for PNIPAAm97-*co*-PAA3), the LCST of the copolymer increased slightly from 30.8 °C at pH 3.0 to 32.8 °C at pH 4.5, based on UV–Vis spectroscopic analysis, close to that of the PNIPAAm homopolymer. A further increase in the PAA composition to that of PNIPAAm80-*co*-PAA20 caused the LCST to change significantly, from 26.6 °C at pH 3.0 to 43.5 °C at pH 4.5, implying that the PAA content in the random copolymer did affect the LCST in a manner dependent on the pH. The LCSTs decreased significantly upon decreasing the pH for high-PAA-content copolymers, as revealed in Figure 6D and Figure 7D. We attribute this behavior to the higher fraction of intermolecular hydrogen bonds between the PNIPAAm and PAA segments in the random copolymers having higher PAA contents (Figure 4C), hindering the inter-association between the amide groups of PNIPAAm and the H_2_O molecules. As a result, the random copolymer would become more hydrophobic in the aqueous solution, inducing a lower LCST, as had been proposed previously [35]. In contrast, the higher LCSTs were found at higher pH for random copolymers having higher PAA contents (Figure 6D and Figure 7D). As mentioned above, the COOH groups of the PAA segments dissociated into COO^−^ units at higher pH, thereby increasing the solubility (i.e., the hydrophilicity) in aqueous solutions; as a result, we would expect higher LCSTs for random copolymers having higher PAA contents. Figure 8 summarizes the visual phase changes of 5 wt % of PNIPAAm80-*co*-PAA20 aqueous solutions at different temperatures and values of pH. The possible chain conformations corresponding to the visual phase changes in Figure 8 are presented schematically in Scheme 4.

### 3.4. LCSTs and Values of T_g_ of PNIPAAm-co-PAA under CO_2_ and after scCO_2_ Treatment

**Figure 9:**
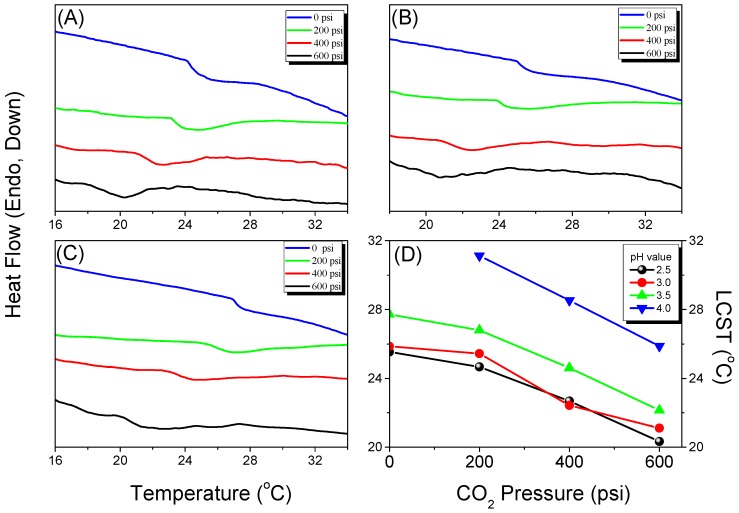
(**A**–**C**) HP-DSC first-heating curves (2 °C/min) of 5 wt % solutions of: (**A**) PNIPAAm97-*co*-PAA3; (**B**) PNIPAAm87-*co*-PAA13; and (**C**) PNIPAAm80-*co*-PAA20 random copolymers at various CO_2_ pressures. (**D**) Corresponding LCSTs of the PNIPAAm80-*co*-PAA20 aqueous solutions at various values of pH.

**Scheme 5:**
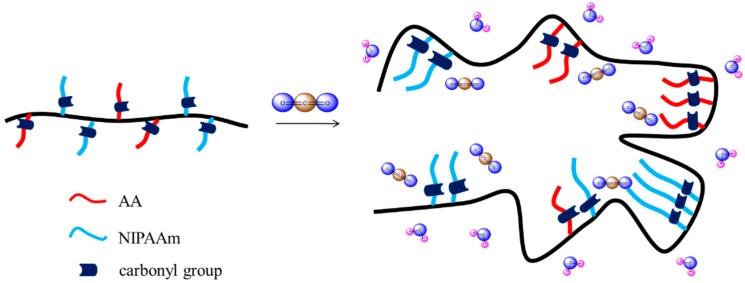
Possible interactions in PNIPAAm-*co*-PAA random copolymers under a CO_2_ atmosphere.

**Figure 10:**
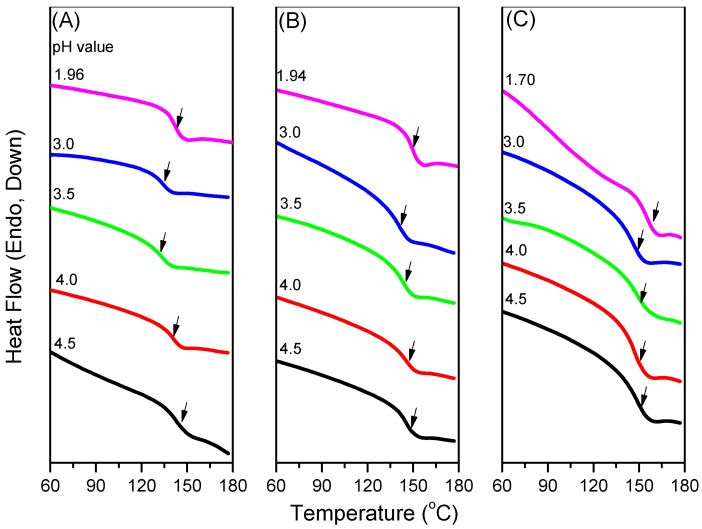
DSC thermograms of: (**A**) PNIPAAm97-*co*-PAA3; (**B**) PNIPAAm87-*co*-PAA13; and (**C**) PNIPAAm80-*co*-PAA20 films cast from aqueous solutions at various values of pH.

**Figure 11:**
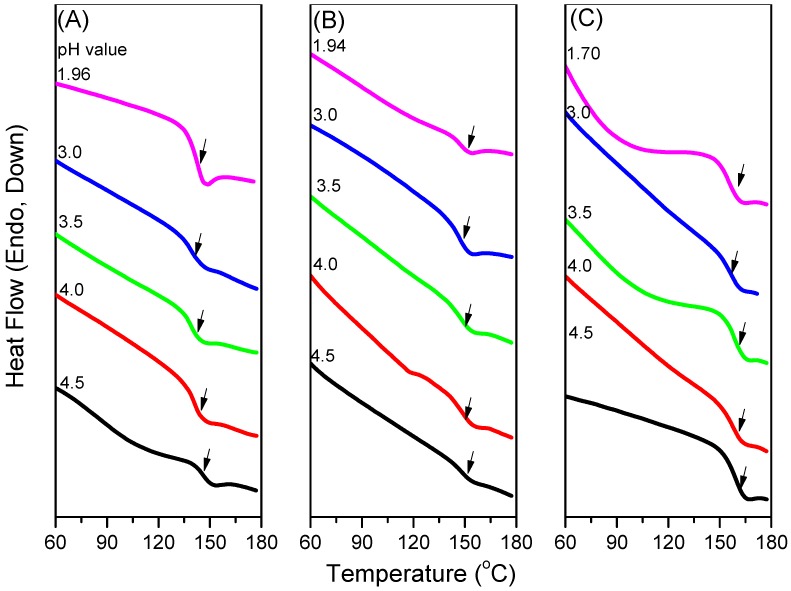
DSC thermograms of: (**A**) PNIPAAm97-*co*-PAA3; (**B**) PNIPAAm87-*co*-PAA13; and (**C**) PNIPAAm80-*co*-PAA20 films cast from aqueous solutions at various values of pH and after treatment in scCO_2_ at 2000 psi and 32 °C for 1 h.

**Figure 12:**
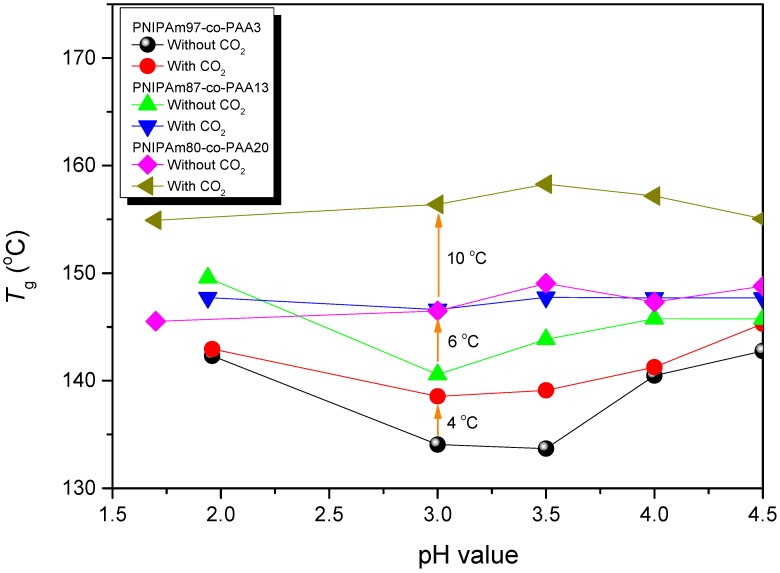
Values of *T*_g_ of PNIPAAm-*co*-PAA films cast from aqueous solutions at various values of pH, with or without scCO_2_ treatment.

Because CO_2_ can interact with the C=O groups of PNIPAAm and PAA through weak dipole–dipole interactions, we used the first heating scans of HP-DSC analyses to investigate the effects of the CO_2_ pressure on the LCSTs of aqueous PNIPAAm-*co*-PAA solutions (Figure 9A–D). The endothermic peaks corresponding to the LCSTs shifted to lower temperatures upon increasing the CO_2_ pressure for all three PNIPAAm-*co*-PAA copolymers, consistent with CO_2_ molecules displacing H_2_O molecules around the polar CONH and COOH groups in the aqueous solutions of PNIPAAm-*co*-PAA^23^. This displacement of H_2_O would enhance the intramolecular hydrogen bonding of the COOH and CONH groups (Scheme 2a,b) and the intermolecular hydrogen bonding of the CONH and COOH groups (Scheme 2c) in the PNIPAAm-*co*-PAA random copolymers, thereby enhancing the hydrophobicity in the aqueous solutions and decreasing in the LCSTs. In addition, the dissolved CO_2_ might form carbonic acid to decrease the pH of the aqueous solutions, thereby decreasing the LCSTs, as discussed above. Scheme 5 summarizes the possible intermolecular interactions of the PNIPAAm-*co*-PAA random copolymers in aqueous solutions under a CO_2_ atmosphere. Figure 9D summarizes the LCSTs of the PNIPAAm80-*co*-PAA20 random copolymer in aqueous solutions of various values of pH, measured under different CO_2_ pressures. In all cases, the LCSTs of the copolymer in the aqueous solutions decreased upon increasing the CO_2_ pressures or decreasing the pH, as discussed previously. Figure 10 summarizes the DSC thermograms, recorded in the range 60–180 °C, of thin films cast from PNIPAAm-*co*-PAA aqueous solutions at various values of pH. All of the films derived from aqueous PNIPAAm-*co*-PAA solutions at values of pH greater than 3 gave lower values of *T*g. For all of the random copolymers, the value of *T*g increased upon increasing the pH of the aqueous solution above pH 3. As mentioned in Scheme 3a, a lower pH would enhance the intermolecular hydrogen bonding between the PNIPAAm and PAA segments, thereby increasing the value of *T*g. As the pH increased, the COOH groups of the PAA segments partially dissociated into COO^–^ units (Scheme 3b), thereby decreasing the strength of intermolecular hydrogen bonding. The negative charge of the COO^–^ units would induce repulsive forces and increase the free volume, leading to a decrease in the value of *T*g. Further increasing the pH would cause the COOH groups of the PAA segments to fully dissociate into COO^–^ units (Scheme 3c). The much greater repulsive forces of the greater number of negatively charged COO^–^ units would result in a rigid structure for the PAA segments; therefore, the value of Tg would increase. Figure 11 summarizes the DSC thermograms of those films in Figure 10 after additional treatment in scCO_2_ at 2000 psi and 32 °C for 1 h, with a depressurization time of 1 h. The values of *T*g of the samples prepared at each value of pH increased after the scCO_2_ treatment, suggesting that it promoted intermolecular hydrogen bonding [36]. Kramer et al. reported that the hydrostatic pressure may increase the value of *T*g by decreasing the free volume of a polymeric material when the pressure environment is soluble in the polymer matrix. In contrast, it may decrease the value of *T*g, through a plasticizer effect, when the pressure environment is not soluble in the polymer matrix [36]. Because CO_2_ can interact with the C=O groups of the PNIPAAm and PAA segments through weak dipole–dipole interactions, and also act as a Lewis acid, the scCO_2_ treatment would promote the formation of COOH groups from COO^–^ units in the PAA segments, thereby facilitating intermolecular hydrogen bonding between the PNIPAAm and PAA segments, as displayed in Scheme 3a, leading to an increase in *T*g, as summarized in Figure 12. This increase in the value of Tg after scCO_2_ treatment was dependent on the PAA content. For example, for the random copolymer PNIPAAm97-*co*-PAA3, the value of *T*g increased by 4 °C, whereas for PNIPAAm80-*co*-PAA20 it increased by 10 °C, after scCO_2_ treatment at pH 3.

## 4. Conclusions

We have synthesized PNIAAm-*co*-PAA random copolymers of different compositions through free radical copolymerizations. Significant increases in *T*_g_ values occurred after incorporation of AA units into PNIPAAm, due to the formation of strong intermolecular hydrogen bonding between the amide groups of PNIPAAm and the carboxyl groups of PAA based on ^1^H NMR and FTIR spectroscopic analyses. The LCSTs of PNIPAAm-*co*-PAA random copolymers were increased upon increasing the pH value because the COOH groups of the PAA segments dissociated into COO^−^ units, thereby enhancing the solubilities of the copolymers in aqueous solutions. HP-DSC revealed that the LCST values of the copolymers decreased after applying CO_2_ pressure, implying that the CO_2_ molecules displaces some of the H_2_O molecules around the polar CONH and COOH groups in the PNIPAAm-*co*-PAA copolymers, thereby enhancing the hydrophobicity of the copolymers in the aqueous solutions. The *T*_g_ values of the copolymers increased after treatment with scCO_2_, implying that intermolecular hydrogen bonding had been enhanced.

## Figures and Tables

**Table 1:** Characteristics of the PNIPAAm-*co*-PAA copolymers used in this study.

Abbreviation	Monomer feed (mol %)		Polymer composition (mol %)		*T*_g_ (°C)	*M*_n_ (g/mol)	PDI
	NIPAAm	AA	NIPAAm	AA			
PNIPAAm	100	0	100	0	140.6	3.1 × 10^5^	1.64
PNIPAAm97-*co*-PAA3	95	5	96.9	3.1	142.3	4.2 × 10^6^	1.70
PNIPAAm87-*co*-PAA13	85	15	86.4	13.6	149.5	2.1 × 10^6^	2.24
PNIPAAm80-*co*-PAA20	75	25	79.6	20.4	154.5	6.8 × 10^6^	1.31
PNIPAAm57-*co*-PAA43	50	50	57.3	42.7	159.1	5.0 × 10^6^	1.56
PNIPAAm30-*co*-PAA70	25	75	29.2	70.8	140.0	8.9 × 10^6^	1.59
PAA	0	100	0	100	88.8	3.1 × 10^5^	1.64
